# The Effects of Emotional Working Memory Training on Trait Anxiety

**DOI:** 10.3389/fpsyg.2020.549623

**Published:** 2021-01-13

**Authors:** Gabrielle C. Veloso, Welison Evenston G. Ty

**Affiliations:** ^1^Department of Psychology, Macquarie University, Sydney, NSW, Australia; ^2^Department of Psychology, Ateneo de Manila University, Quezon City, Philippines

**Keywords:** anxiety, trait anxiety, emotion regulation, working memory, emotional working memory training

## Abstract

**Background:**

Trait anxiety is a pervasive tendency to attend to and experience fears and worries to a disproportionate degree, across various situations. Decreased vulnerability to trait anxiety has been linked to having higher working memory capacity and better emotion regulation; however, the relationship between these factors has not been well-established.

**Objective:**

This study sought to determine if participants who undergo emotional working memory training will have significantly lower trait anxiety post-training. The study also sought to determine if emotion regulation mediated the relationship between working memory training and trait anxiety.

**Method:**

An experimental group comprising of 49 participants underwent 20 days of computerized emotional working memory training, which involved viewing a continuous stream of emotionally-charged content on a grid, and then remembering the location and color of items presented on the grid. The control group comprised of 51 participants.

**Results:**

Participants of the experimental group had significantly lower trait anxiety compared to controls, post-training. Subsequent mediation analysis determined that working memory training capacity gains were significantly related to anxiety reduction as measured by form Y2 of the Spielberger State-Trait Anxiety Inventory (STAI-Y2). Emotion regulation, as measured by the Emotional Regulation Questionnaire (ERQ), was found not to mediate between working memory capacity gains and trait anxiety reduction.

**Conclusion:**

Working memory capacity gains and reductions in levels of trait anxiety were observed following emotional working memory training. The study may therefore be useful in informing interventions targeted at improving working memory capacity, and reducing levels of trait anxiety. Moreover, it proposes for future research to further look into the mediating role of emotion regulation via the development or utilization of more comprehensive measures of emotion regulation.

## Introduction

Emotional working memory training is a working memory training program that makes use of emotionally-charged stimuli for exercises aimed at increasing working memory capacity (Mammarella, 2014). The role of working memory training programs, including emotional working memory training, in reducing anxiety is a recent and understudied topic in research that is also the source of debate. There has been conflicting evidence regarding the efficacy of working memory training on improving affect. Some research suggests that training working memory can reduce negative affect (Roughan and Hadwin, 2011; Schweizer et al., 2011, 2013; Franken, 2014; Takeuchi et al., 2014; Sari et al., 2015; Hadwin and Richards, 2016; Beloe and Derakshan, 2019), whereas other studies have found that training working memory showed no significant impact on affect (Onraedt and Koster, 2014; Wanmaker et al., 2014, 2015; Leone de Voogd et al., 2016). Moreover, few studies have explored the link between working memory training and trait anxiety, in particular. Trait anxiety is characterized by a pervasive tendency to experience symptoms of anxiety (Gellman, 2012). Trait anxiety would therefore remain to be stable unless influenced by external factors such as interventions (Gellman, 2012). With regard to working memory training targeted at reducing trait anxiety, studies that have tackled this include the works of Sari et al. (2015); Hadwin and Richards (2016), and Beloe and Derakshan (2019), who found that working memory training was associated with lower levels of trait anxiety or vulnerability to trait anxiety, and the works of Wanmaker et al. (2015) and Leone de Voogd et al. (2016), who found no significant difference in anxiety levels between experimental and control groups. These studies are also limited by factors such as the absence of an inactive control group (Hadwin and Richards, 2016), significant differences found only within specific individuals and not between groups (Sari et al., 2015), a high drop-out rate (Wanmaker et al., 2015), the use of anti-depressants in the placebo group (Wanmaker et al., 2015), insufficient training (Leone de Voogd et al., 2016), and baseline symptomology in participants being too low to be significantly reduced by the training (Wanmaker et al., 2014). That said, it is evident that the few studies linking working memory training and trait anxiety have not yet produced a conclusive answer to the question regarding the effects of working memory training on trait anxiety. This study sought to contribute to the ongoing discussion by examining the efficacy of emotional working memory training in reducing levels of trait anxiety, while also introducing a possible mediating factor: emotion regulation.

### Literature Review

Trait anxiety is a pervasive tendency to attend to, experience, and report negative emotions such as fears and worries across various situations (Gellman, 2012). There is strong evidence that trait anxiety significantly and negatively impacts individuals psychologically, physiologically, and functionally (Culpepper and Conner, 2004). It is believed that because trait anxiety is chronic in nature, these negative experiences continue to persist unless the cognitive processes that perpetuate trait anxiety are targeted and re-trained in intervention (Williams et al., 1988). Among these cognitive processes, of interest are attentional bias and control, emotion regulation, and working memory capacity.

It has been strongly suggested that attentional bias toward threat and attentional control processes underlie trait anxiety (Williams et al., 1988). Williams et al. (1988) found that individuals with high trait anxiety allocate more attention to threat as compared to people with low trait anxiety (Williams et al., 1988). Moreover, individuals with high trait anxiety maintain their attention toward threatening stimuli significantly longer than do individuals with low trait anxiety (Williams et al., 1988). A viable explanation for this would be that individuals with trait anxiety have less attentional control resources to be able to minimize disruption, interference, and fixation from irrelevant but emotionally-charged stimuli (Veerapa et al., 2020). Indeed, impaired attentional processes have been identified as among the primary cognitive factors underlying not only the development, but also the maintenance of trait anxiety (Eysenck et al., 2007).

Another related cognitive process that is believed to be reduced in highly trait anxious individuals is emotion regulation (Kashdan et al., 2008; Cisler et al., 2010). Emotion regulation is defined as a set of actions used to modulate which emotions are experienced, when they are experienced, and how they are experienced and expressed (Cisler et al., 2010). While emotion regulation is often characterized as a broad range of cognitive mechanisms, for the purpose of this paper, emotion regulation will be operationally defined as a specific subgroup of these cognitive mechanisms that rely on attentional processes subsumed by working memory, namely, distraction, concentration, and updating. These emotional regulation strategies are believed to be pertinent in preventing and diminishing experiences of anxiety (Cisler et al., 2010; Mammarella, 2014).

*Distraction* means shifting attention from one affective content to another (Mammarella, 2014). As stated, highly trait anxious individuals tend to sustain their attention toward threatening stimuli for longer periods, thereby increasing their experience of anxiety (Williams et al., 1988). Distraction is an emotion regulation mechanism by which individuals shift their focus of attention away from threatening content to prevent feelings of anxiety. For example, an individual might redirect their attention to a more neutral or positive thought in the face of irrelevant negative stimuli. This can be helpful in reducing negative responses (Mammarella, 2014). *Concentration* refers to sustaining attention on a specific content or task (Mammarella, 2014). Highly trait anxious individuals have attentional biases toward threat, and are more likely to shift their attention away from the present task, and toward irrelevant negative stimuli (Williams et al., 1988). In contrast, a person who is able to sustain their attention on a specific object or task despite interfering negative stimuli, is able to prevent such stimuli from intruding on their present cognitive and emotional state (Mammarella, 2014). *Updating* means transforming short-term information by first retrieving this information, deliberately perceiving it in a different way, and then substituting the previous perception with the most recent one (Ecker et al., 2010). For example, an individual who perceives a stimuli as anxiety-provoking might transform their interpretation of this to one that is less threatening. The emotion regulation strategies of distraction, concertation, and updating are believed to require internal resources, namely, working memory capacity (Gyurak et al., 2012; Opitz et al., 2012).

Working memory capacity is a broad term that is operationalized in various ways across different research fields (Wilhelm et al., 2013). For the purpose of this study, working memory capacity is defined as the ability to control and sustain one’s attention for the purpose of holding and manipulating information in the face of interference or distraction (Engle, 2002; Gyurak et al., 2012; Opitz et al., 2012). Recent studies have suggested that working memory capacity, which subsumes attentional control, is significantly associated with emotion regulation ability (Gyurak et al., 2012; Opitz et al., 2012). In the Selection, Optimization, and Compensation with Emotion Regulation (SOC-ER) framework, working memory capacity is believed to be the among most frequently utilized internal resources used to regulate one’s emotions (Opitz et al., 2012). Indeed, being able to shift the focus of one’s attention within working memory via distraction (Berti, 2016) and focus on target-relevant information while eliminating responses to distractors and interferences via concentration (Schmeichel et al., 2008) are emotion regulation strategies that are suggested to depend heavily on working memory capacity (Miyake et al., 2000). Additionally, the ability to update or manipulate affective information in working memory such that negative interpretations and reactions are reduced (Kensinger and Corkin, 2003; Cisler et al., 2010; Pe et al., 2013; Mammarella, 2014) is also believed to be dependent on working memory capacity (Miyake et al., 2000).

The effectiveness of working memory training on the aforementioned emotion regulation strategies has been explored by Xiu et al. (2018). Participants completed a working memory task in a 20 day training period that required employing working memory to different stimuli such as letters, animals, and locations. Emotion regulation was measured via a task wherein participants were presented neutral and negative stimuli while they performed the appropriate task: Watch (neutral images), Attend (negative images), Distract (negative images), or Reappraise (negative images). EEG data were simultaneously recorded. Late positive potential (LPP) was used to assess working memory training’s effect on emotion regulation (Xiu et al., 2018). LPP is an is a scalp-recorded event-related potential (ERP) that reflects attention to emotional stimuli (Dennis and Hajcak, 2009). The magnitude of LPP is larger when individuals view emotionally arousing images as compared to neutral images (Dennis and Hajcak, 2009). Research has found that LPP magnitude in adults are decreased when individuals employ emotion regulation strategies (Foti and Hajcak, 2008). For this reason, the researchers used reduced LPP magnitude as an indicator for improved emotion regulation (Xiu et al., 2018). After the 20 day training period, it was found that there was significant difference in the Distract and Reappraise conditions, with the LPP amplitude of the training group being less than that of the control group (Xiu et al., 2018). This suggest that working memory training may have improved emotion regulation; particularly with regards to distraction and reappraisal (Xiu et al., 2018).

Conversely, a study by Lee and Xue (2018) that sought to determine if emotion regulation is mediated by working memory via a meta-analytical study of published neuroimaging literature, found contradicting evidence. The neural circuits that regulate negative emotion, particularly, reappraisal, and working memory, were found to both activate the dorsal midline prefrontal cortex (Lee and Xue, 2018). However, peak coordinates of emotion regulation were in the middle frontal cortex, which was dorsal to those of working memory by 15.1 mm to the left and 21.6 mm to the right (Lee and Xue, 2018). These findings may negate the assumption that reappraisal (updating) and working memory rely on the same neural circuits (Lee and Xue, 2018). Given this conflicting evidence, further investigation is necessary to determine if emotion regulation relies on working memory capacity, at least in the case of updating. This study sought to do so by examining the links between emotional working memory training, emotion regulation, and trait anxiety.

Emotional working memory training (EWMT) focuses on targeting working memory capacity, particularly in how affective information is attended to and manipulated (Mammarella, 2014). It is believed that distraction, concentration, and updating can be trained in order improve the regulation of emotions, thereby decreasing anxiety (Mammarella, 2014). There are only a few studies that have sought to determine the effects of working memory training on anxiety, and the results are mixed. One study by Beloe and Derakshan (2019) found that 20 days of adaptive working memory training reduced vulnerability to anxiety in adolescents with sub-clinical symptomology, and results were sustained after a 1 month follow-up. However, the study made use of the Revised Child Anxiety and Depression Scale (RCADS), which collates symptoms of separation anxiety disorder, social phobia, generalized anxiety disorder, panic disorder, obsessive compulsive disorder, and major depressive disorder (Beloe and Derakshan, 2019). That said, the effect of the training on trait anxiety in isolation was not specified.

A study by Sari et al. (2015) examined the effects of working memory training on attentional control in high trait anxious individuals who underwent a 3 week daily working memory training. The training program made use of a grid-like game field in which users are to remember and match locations and words simultaneously. After the intervention, it was found that the training group did not have significantly reduced anxiety at post-training compared to the control group; however, researchers found decreased anxiety scores for participants who improved the most on the training task and were highly engaged (Sari et al., 2015). Another study by Hadwin and Richards (2016) utilized working memory training intervention for their experimental group, and an active cognitive behavioral therapy (CBT) control group. Their working memory training consisted of eight computerized tasks involving visuo-spatial or verbal working memory (Hadwin and Richards, 2016). Participants underwent 15 trials per session, with increasing number of items to be remembered per trial (Hadwin and Richards, 2016). Twenty-five sessions, 5 days per week were completed (Hadwin and Richards, 2016). After the intervention period, both groups reported fewer anxiety symptoms with no significant difference between groups, which suggests that working memory training has similar benefits as CBT on trait anxiety (Hadwin and Richards, 2016). However, the study did not use an inactive control group.

In contrast to findings in previously mentioned research, a study conducted by Leone de Voogd et al. (2016) found that working memory training was not effective in reducing anxiety. The researchers assigned participants over an active or placebo emotional working memory training over 4 weeks of bi-weekly training. Both conditions involved viewing a fixation cross, followed by a 4 × 4 matrix of green and blue squares that would light up in a particular sequence (Leone de Voogd et al., 2016). Participants had to reproduce this sequence by clicking these squares in the correct order as presented (Leone de Voogd et al., 2016). The emotional component of the training consisted of a negative emotional face that presented on one of the squares, and had to be omitted in the reproduction of the sequence (Leone de Voogd et al., 2016). The difference between the conditions was that the active working memory training increased in difficulty depending on the participant’s performance, while in the placebo condition, difficulty was not increased (Leone de Voogd et al., 2016). The researchers found, post intervention, that there was no difference between the active and placebo groups with regards to symptoms from a range of pervasive anxiety disorders. Researchers stated that this may in part be due to the fact that there were only eight training sessions, with limited duration (Leone de Voogd et al., 2016). Similarly, a study by Wanmaker et al. (2015) also produced outcomes that put the effectiveness of working memory training on trait anxiety reduction into question. The researchers conducted a double-blind randomized controlled trial in a sample of 98 patients with anxiety and/or depression, employing working memory training for the purpose of attenuating anxiety and depression levels (Wanmaker et al., 2015). The working memory training was conducted three times a week across 3 weeks (Wanmaker et al., 2015). The training involved a role-play format, wherein participants had characters, and freely choose among a selection of working memory tasks to undertake in order to increase their character’s level of strength (Wanmaker et al., 2015). Difficulty increased with performance (Wanmaker et al., 2015). In the placebo condition, difficulty level was set low and remained unchanged throughout (Wanmaker et al., 2015). The researchers found that the working memory training did not significantly reduce symptoms (Wanmaker et al., 2015). They state limitations such as a high drop-out rate and the use of anti-depressants in the placebo group (Wanmaker et al., 2015).

This study sought to contribute to the discussion, given that the limited research regarding the efficacy of working memory training in reducing trait anxiety appears to be inconclusive as of the present. The study also sought to address some of the limitations posed by previous studies via specification of the type of anxiety being targeted, the use of an inactive control group, ensuring sufficient training sessions, ensuring sufficient sample size, ensuring sufficient baseline levels of anxiety, and screening for the use of medications used to manage mood. Lastly, the study examined specific emotion regulation strategies that may be dependent on working memory capacity, which could potentially add depth to the present understanding of how working memory capacity, emotion regulation, and trait anxiety are interrelated.

## Theory

**FIGURE 1:**
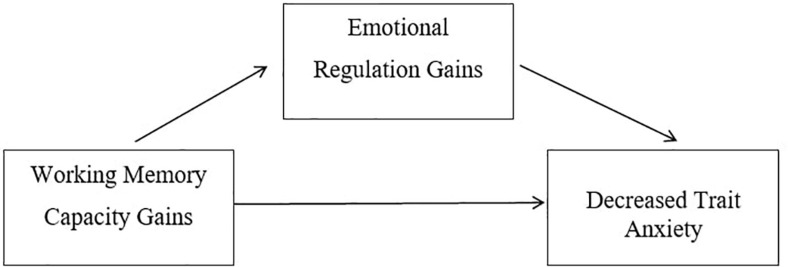
Conceptual framework.

Emotion regulation strategies such as distraction, concentration, and updating, are believed to depend on working memory capacity (Miyake et al., 2000; Pe et al., 2013; Mammarella, 2014). When linked to the work of Cisler et al. (2010), who stated that emotional regulation plays a vital role in alleviating anxiety such that anxious experiences can be augmented or diminished, it can be suggested that increased working memory capacity via emotional working memory training can improve emotion regulation, thus, reduce symptoms of trait anxiety (see Figure 1).

## Materials and Methods

### Design

A pre-test–post-test randomized control experimental design was used to compare the posttest scores of participants belonging to the experimental group from the control group, as well as to determine to what extent emotion regulation mediates the relationship between working memory capacity gains following working memory training, and trait anxiety reduction.

### Participants

The program G^∗^Power was used to determine the recommended sample size for the study, based on an anticipated effect size of 0.36 when comparing scores before and after training. This anticipated effect size is based on similar studies that have found significant effects of emotional working memory training on anxiety (Hadwin and Richards, 2016) and affective control (Schweizer et al., 2011, 2013). A sample size of 102, with 51 participants per condition was recommended to achieve a statistical power of 0.8 with the probability of error at 0.05. One hundred eighty-four participants consented to being part of the study. The participants were recruited in several batches, with each batch being split between experimental and control condition via online random sampling that utilizes a digital version of the fishbowl method. In total, 92 participants were assigned to each group.

However, only 49 participants of the experimental group were able to complete the emotional working memory training while complying to the instructions. Some participants skipped several days of training, did not complete the 20 days of training, or requested to drop out. Their data was therefore excluded from the study. In the control group, only 51 participants completed the post-test within the timeline provided. Others completed the post-test beyond the 20 day interval or not at all, so their data was not included. Overall, there were 100 participants who completed the study.

Participants were aged 18–40 years old (*M* = *26.94, SD* = *5.68*). There was no difference in age between participants of the experimental group (*M* = *25.98, SD* = *5.2*) and control group (*27.86, SD* = *6.02*); [*t*(98) = −1.67, *p* > 0.01]. With regard to gender, 49% of the participants were male, 51% were female, with no significant difference between percentages of males and females between the experimental and control groups X^2^(1) = 0.157, *p* > 0.05).

With regard to inclusion criteria, all participants had a cut-off score of 30 on form Y-2 of the Spielberger State-Trait Anxiety Inventory (STAI), which indicates normal levels of anxiety. This cut-off score was intended to ascertain that even mildly elevated levels of anxiety were present, and could be attenuated by the training. Other inclusion criteria were comfortability with English as a medium of communication given that word stimuli in the training program are in English, not currently undergoing psychiatric medication, in order to reduce confounding effects, and lastly, computer literacy as well as owning a personal computer or laptop.

Prior to conducting the research, an ethics review approval was obtained from a University-based Ethics Review Board. An ethical issue anticipated was the concern for anonymity of data. This was addressed by a section in the informed consent form that explained to participants that their data will only be known to the researcher. Moreover, names were replaced with a number, in order to further guarantee confidentiality. After the data was analyzed, all identifying information about the participants was permanently deleted. Data of participants who dropped out were also permanently deleted.

There were also unique ethical considerations for the two groups in the study. The experimental group was asked to undergo emotional working memory training every day for 20 days. An ethical concern was the potential discomfort the participants may experience due to the emotional content of the training program, as the stimuli in the program includes facial expressions as well as words, some of which are emotionally-charged (e.g., rape, sick, hate). While all studies that have made use of the training program in the past found no reports of psychological discomfort, the risk was still addressed. In order to control for this risk, the inclusion criteria prohibited people who are under the age of 18 to participate. This was done to ensure that participants would be of some emotional maturity and would be able to consent in full knowledge of the risks involved, which was explained in the informed consent form. They were also told in the informed consent form that they could discontinue at any time, and if they felt any sort of discomfort, they could contact the researcher, who would then refer them to a psychologist. Another concern was that the amount of time these participants were asked to invest in the program may not be deemed worthwhile, especially if the training gains were found to lack significance. That said, participants were properly compensated. Individuals belonging to the experimental group were given a fixed amount of monetary compensation worth 30 USD for their time spent on the training program, similar to the works of Schweizer et al. (2013) and Takeuchi et al. (2014). Participants of the control group were compensated minimally with 5 USD, given that their condition demanded significantly less time commitment. It must be noted that monetary compensation was not anticipated to influence the affect of the participants, as Takeuchi et al. (2014) found no effects of monetary reward as an inducement of mood change. The offer of monetary compensation was made known to participants in the informed consent form.

### Measures

The anxiety scale used as a pre-test and post-test as well as the screening measure was form Y-2 of the Spielberger State-Trait Anxiety Inventory (STAI), which measures trait anxiety. The Emotional Regulation Questionnaire (ERQ) was used to measure respondents’ tendency to regulate their emotions. Subscales include cognitive reappraisal and expressive suppression. A working memory capacity test, the Automated Operation Span Task (OSPAN), was also used.

Form Y-2 of the STAI accounts for stable aspects of anxiety proneness, or trait anxiety (Spielberger, 1989). Form Y-2 was utilized since the study aims to decrease symptoms of trait anxiety in particular. Scores of the scale range from 20 to 80. The scale measures symptoms of anxiety with statements such as “I lack self-confidence” and “I feel rested” to be rated on a Likert scale. A study that used the said measure among engineering students yielded a KMO of 0.783, *p* < 0.01; and a Cronbach alpha of 0.78, indicating acceptable reliability (Vitasari et al., 2011). Test-retest reliability coefficients of the entire STAI scale range from 0.65 to 0.75 (Spielberger et al., 1983). With regards to validity, the scores obtain from the use of the scale correlated with two widely used measures of trait anxiety, the IPAT Anxiety Scale and the Taylor Manifest Anxiety Scale (TMAS) (Spielberger et al., 1983). Correlations between the trait anxiety scale of the STAI and the IPAT, as well as the TMAS were relatively high, ranging from 0.85 to 0.73 (Spielberger et al., 1983).

The Emotional Regulation Questionnaire (ERQ) is a 10-item scale that measures respondents’ tendency to regulate their emotions through cognitive reappraisal, which is characterized by updating (Kashdan et al., 2008; Pe et al., 2013), as well as expressive suppression, which is believed to be dependent on distraction and concentration mechanisms (Enebrink et al., 2013). Scores range from 10 to 70. A higher score on the scale indicates a greater use of emotion regulation strategies. The scale was found to have adequate internal consistency, with the Cronbach’s alpha of cognitive reappraisal scale being 0.81 and 0.73, for the expressive suppression scale (Enebrink et al., 2013). Test-retest reliability across 3 months was 0.69 (Gross and John, 2003). Criterion validity showed cognitive reappraisal to be related to greater positive affect (*r* = 0.42), mood repair (*r* = 0.36), and life satisfaction (*r* = 0.30), as well as to reduced negative affect (*r* = −0.51) and depression (*r* = −0.23 to −0.29) (Gross and John, 2003).

A working memory capacity test, the Automated Operation Span Task (OSPAN), was used to measure working memory capacity. The OSPAN asks users to solve simple math problems, while remembering a letter flashed in the screen between each math problem. After each set of math problems, with the amount of problems and letters increasing for every set, users are to input the letters that were flashed in their screen in the order that they appeared. The entire test takes 30 min long, inclusive of the initial tutorial. The test was found to be a reliable and valid measure of working memory (Unsworth et al., 2005; Redick et al., 2012). With regard to stability, studies showed that the increase in score upon taking the test for the second time was only two to three items, indicating relatively small practice effects and demonstrating high test-retest reliabilities across all test sessions (Unsworth et al., 2005). Moreover, the item sets are randomly generated, and vary each testing session.

### Material

**FIGURE 2:**
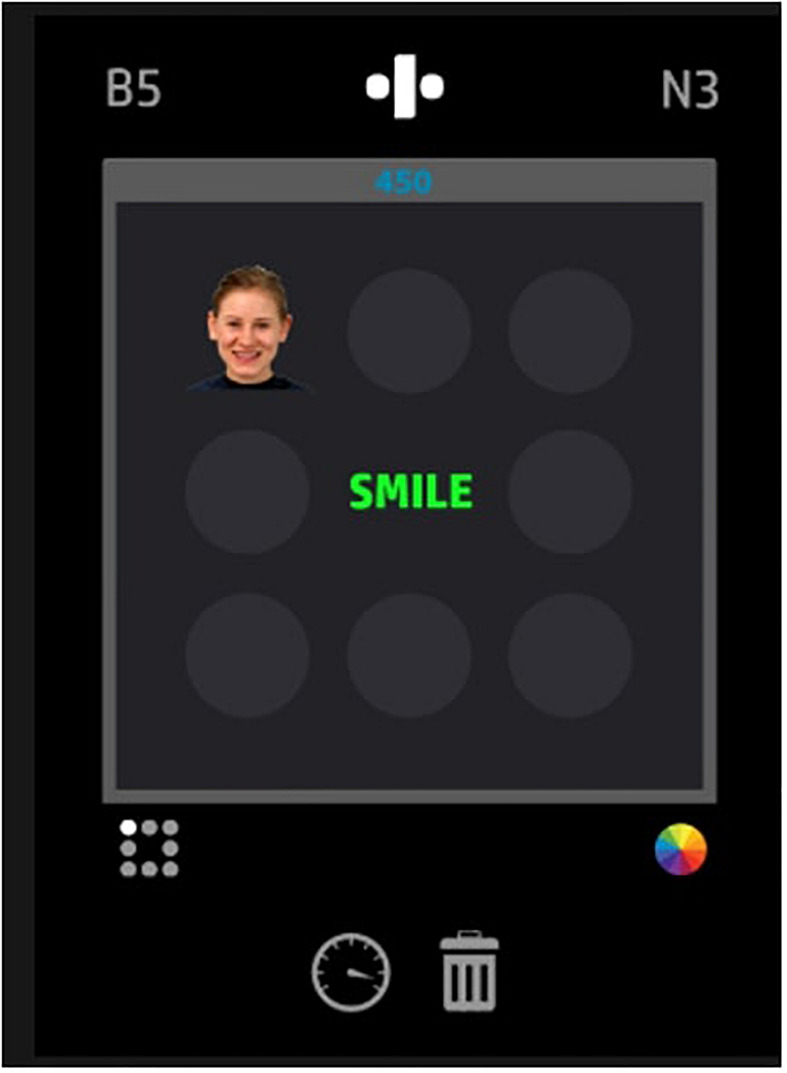
Working memory training game field (Smith, 2016).

The working memory training program used, Emotional Dual n-Back, is one condition that is part of the 2G Dual n-Back working memory training. This specific condition involves viewing a continuous stream of photographs of human models’ facial expressions in varying locations on a 3 × 3 grid, accompanied by a colored word at the center of the grid (see Figure 2). Each picture-word pair is presented in a 2,500 ms interval. The location of the face, as well as the type of face, changes in each interval, as does the color of the word and the actual word presented. The types of faces and words presented as a pair are randomized. Participants need to remember the location of the faces and the color of the words. The actual facial expression and word content are to be ignored. Within each 2,500 ms interval, the participant is to press the F key if the location of the face is the same “*n*” turns ago, and is to press the L key if the color of the word is the same “*n*” turns ago. Here, “*n*” is the number of intervals wherein a picture-word pair appeared. Participants start with an “*n*” of 1, meaning that they need to remember picture-word pairs one interval prior. However, as the difficulty of the game increases in proportion to performance, “*n*” increases. Moreover, the amount of milliseconds in each interval is also reduced as difficulty increases. The training sessions consist of 20 blocks or trials, which make up two “sessions,” and lasts 20 min long. At the end of the sessions, a progress report is displayed on the participant’s screen.

This emotional working memory training relies on a series of working memory functions such as attentional processes, encoding, rehearsal, matching, temporal ordering, inhibition of currently irrelevant items, and response execution (Mammarella, 2014). More importantly, the training makes use of emotionally-charged content for the purpose of targeting emotion regulation strategies that are dependent on working memory. The photographs of facial expressions include neutral faces as well as negative faces (e.g., angry, shouting, scared, crying), and many of the words were chosen specifically to trigger emotional responses (e.g., “money,” “hate,” “sick,” “smile,” “evil,” “prize,” “slum,” “rape,” “dead”). The program prompted participants to rapidly shift their attention away from task-irrelevant emotionally-charged stimuli (i.e., facial expressions, words), toward more neutral task-relevant stimuli (i.e., location, color). Participants also had to ensure that their attention was sustained on the task-relevant information, as opposed to the presenting task-irrelevant emotionally-charged stimuli. Attention shifting and sustained attention that are involved in the emotion regulation strategies of distraction and concentration respectively, are therefore targeted. In addition to this, updating is involved to the extent that participants replace emotional responses and perceptions about the presenting task-irrelevant stimuli with more neutral interpretations that support that these emotionally-charged stimuli are irrelevant.

### Procedure

The STAI form Y2 and the ERQ were administered to individuals through an online survey that was attached to the end of an invitation to participate in the study. Those whose score met the 30-point cut-off on form Y2 were considered as candidates for the study. The cut-off point indicated that the sample experiences some form of anxiety –mild anxiety at the very least. Candidates were given an email with the informed consent form. This included information that there would be an experimental group and a control group, however, candidates would not be made aware of which group they are assigned to until after the completion of the study. Moreover, while all recruitment was conducted online in order to reduce the chances of participants communicating, additional precaution was taken by including a section in the consent form that asks participants to keep the details of their condition confidential until after the study is completed. Those who signed the form were randomly assigned to either the working memory training group, or the inactive control group. Participants were given an email that explained what is to be expected of them, depending on their assigned group.

Participants of the experimental group first took the OSPAN online. They were then given a link to download the emotional working memory training program installer, which they then installed in their personal devices, as well as instructions on how to use the program. They then used the training program for 20 min a day, daily, for 20 days. Participants underwent the training at their own time but maintained the schedule of 20 min every day for 20 days. Those who did not adhere to the schedule were considered “dropped.” Evidence of each training session per participant was collected in order to ensure that the program was followed religiously. This was done by having the participants take screen shots of the training at the beginning and end of each session. Screen shots provided evidence of compliance because the elapsed time was visible, and the game displayed how many “blocks” or game sets a participant has completed. If a participant was inactive throughout their 20 min of training, the time shown on the screenshot would demonstrate that 20 min had elapsed, however, an incomplete number of “blocks” would be displayed. Moreover, the participant’s scores across all blocks are graphed to show their performance. This not only provided evidence that all blocks per training day were completed in 20 min as required, but also showed if the participant was active throughout their session based on the pattern of their scores. The screenshots were collected from the participants at the end of the 20 day period via email, as agreed upon in the consent form. However, most participants chose to send their screenshots daily because they believed this to be more helpful in maintaining their routine. Weekly email reminders and reminders through Short Messaging Service (SMS) were sent to participants.

Participants of the control group did not undergo any intervention. They were simply told to practice healthy habits throughout the 20 day period.

After the 20 day training period, participants of both groups were re-administered the STAI form Y2 and the ERQ online. Participants of the experimental group were additionally given the OSPAN once again, after completion of the questionnaire. After which, all scores were analyzed. Participants were debriefed via email and were given the results of their questionnaire with adequate explanation, upon request.

### Data Analysis

An independent samples *t*-test test was first used to determine if the experimental group and the control group had varying levels of STAI and ERQ scores at baseline. Another independent samples *t*-test test was used to determine if the experimental group showed significantly lower scores on the STAI and significantly higher scores on the ERQ at the post-test, as compared with the control group. Within-subjects *t*-tests were used to determine if there were significant changes in STAI, ERQ, and OSPAN scores from pre-test to post-test in the experimental group, and if there were significant changes in STAI and ERQ scores from pre-test to post-test in the control group.

In addition, two by two analysis of variance (ANOVA) was then used to determine main effects and interaction effects of changes in STAI and ERQ scores from pre-test to post-test, and condition (i.e., working memory training vs. no training). ANOVAs for STAI scores and ERQ scores were conducted separately.

Simple mediation analysis using PROCESS by Andrew F. Hayes was conducted to determine if emotion regulation gains mediated the relationship between working memory capacity gains and reduced trait anxiety. Emotional regulation gains were measured by ERQ score differences pre and post training. Working memory capacity gains were measured by OPSAN score differences pre and post training. Anxiety reduction was measured by STAI score differences pre and post training. Bootstrapping was applied in order to ensure that all test assumptions have been met.

## Results

For the purpose of this section it must also be noted that lowered or reduced scores on the STAI indicate reduced levels of trait anxiety, and increases or higher scores in the OSPAN and ERQ indicate working memory capacity gains and emotion regulation gains respectively.

At baseline, there was no significant difference between experimental and control groups in their anxiety levels as measured by the STAI [*t*(98) = −1.16 *p* > 0.05], and in their emotion regulation as measured by the ERQ [*t*(98) = 0.97, *p* > 0.05]. This provided for appropriate comparisons to be made between the groups.

### Pre-test Post-test Comparisons

Data analysis showed that in the experimental group, there was a significant increase in the scores of the OSPAN from pre-test to post-test [*t*(48) = −6.88, *p* < 0.01]. See Table 1.

**TABLE 1:** OSPAN scores for experimental group.

	Experimental group	
Measurement	*M*	*SD*
Pre-test	51.97	16.09
Post-test	63.67	13.30

With regard to the STAI, data analysis showed that STAI post-test scores were significantly lower in the experimental group compared to the control group [*t*(98) = −5.18, *p* < 0.01]. For the experimental group, there was also a significant decrease in the scores of the STAI from pre-test to post-test [*t*(48) = 8.12, *p* < 0.01]. This was not observed in the control group, in which no significant difference in the scores of the STAI from pre-test to post-test were observed [*t*(50) = −0.96, *p* > 0.05].

There was a significant interaction effect between changes in STAI scores and condition [*F*(1,196) = 8.55, *p* < 0.01]. The main effect of pre-test to post-test differences was significant [*F*(1,196) = 6.89, *p* < 0.01], as was the main effect of condition on ERQ scores [*F*(1,196) = 20.50, *p* < 0.01]. See Table 2.

**TABLE 2:** STAI pre-test and post-test scores by group.

	Experimental group		Control group	
Measurement	*M*	*SD*	*M*	*SD*
Pre-test	44.55	7.01	46.54	9.97
Post-test	37.63	8.84	46.92	9.08

With regard to the ERQ, data analysis showed that there were significantly higher post-test scores in the experimental group as compared to the control group [*t*(98) = 1.66, *p* < 0.05]. It was also found that there was a significant increase in the scores of the ERQ from pre-test to post-test [*t*(48) = −4.15, *p* < 0.01] in the experimental group. However, these gains were also found in the control group, with significant increases in ERQ scores from pre-test to post-test [*t*(50) = −3.91, *p* < 0.01].

The interaction effect between ERQ pre-test to post-test changes and condition showed to be not significant [*F*(1,196) = 0.25, *p* > 0.05]. The main effect of pre-test to post-test differences were significant [*F*(1,196) = 4.69, *p* > 0.05], however, the main effect of the condition on ERQ scores was not significant [*F*(1,196) = 3.49, *p* > 0.05]. See Table 3.

**TABLE 3:** ERQ pre-test and post-test scores by group.

	Experimental group		Control group	
Measurement	*M*	*SD*	M	*SD*
Pre-test	45.36	8.84	43.45	10.74
Post-test	49.08	10.11	45.78	9.62

### Mediation Analysis

**FIGURE 3:**
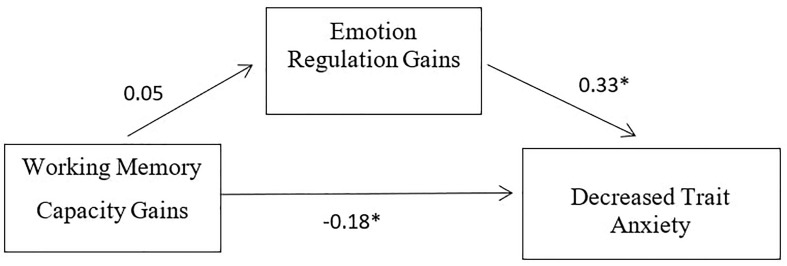
Mediation analysis results.

The total effect of the model was significant [*B* = −0.16, *t*(47) = −2.19, *p* < 0.05]. The direct effect of working memory capacity gains on anxiety reduction was significant [*B* = −0.18, *t*(46) = −2.64, *p* < 0.01]. However, the indirect effect was 0.02, and the 95% confidence interval ranged from −0.03 to 0.05, which proved to be not significant. The effect of working memory capacity gains on emotion regulation was not significant [*B* = 0.05, *t*(47) = 0.59, *p* > 0.05], while the effect of emotion regulation gains in anxiety reduction was significant [*B* = 0.33, *t*(46) = 3.14, *p* < 0.01]. See Table 4 for *R*-squared values and Figure 3 for a summary of the mediation analysis results.

**TABLE 4:** Measure of effect of mediator variable.

Model	*R* squared	*R* squared change	%
Model with working memory capacity gains	0.0923	–	–
Model with working memory capacity gains + emotion regulation gains	0.2524	0.1601	16.01

## Discussion

The efficacy of working memory training in reducing trait anxiety has been a topic of much debate in the recent literature (Roughan and Hadwin, 2011; Schweizer et al., 2011, 2013; Franken, 2014; Onraedt and Koster, 2014; Takeuchi et al., 2014; Wanmaker et al., 2014, 2015; Sari et al., 2015; Hadwin and Richards, 2016; Leone de Voogd et al., 2016; Beloe and Derakshan, 2019). This study sought to contribute to the discussion, while addressing some of the limitations stated by previous studies. The study initially recruited a large sample size in order to account for participant drop-outs, and also utilized an inactive control group. Additionally, it was ensured that baseline levels of anxiety were elevated enough to reflect statistically significant reductions following the working memory training. The study also ensured that no participants in either the active and control group were making use of psychiatric medication. Sufficient training sessions based on past recommendations (Leone de Voogd et al., 2016) were also implemented in the experimental group.

The results show that working memory capacity gains were observed in the experimental group following emotional working memory training. The working memory training program used in this study involved dissimilar tasks as compared with the measure used to obtain working memory capacity. That said, some forms of working memory training, such as the one used in this study, might be effective in improving working memory capacity more broadly. This contributes to the debate about whether or not working memory training is effective in improving working memory capacity beyond the training task itself (Turner and Engle, 1989).

With regard to anxiety, it was found that after the 20 day period, trait anxiety levels were significantly lower among participants who underwent the emotional working memory training as compared to controls. After the 20 day period, participants of the control group did not vary in their levels of trait anxiety, whereas those in the experimental group experienced a reduction in trait anxiety. Interaction and main effects of the working memory training also proved to be significant, suggesting efficacy of the training in reducing levels of trait anxiety. These findings were reinforced by results of the mediation analysis, which showed that working memory capacity gains following working memory training were associated to anxiety reduction in the experimental group. Therefore, it can be suggested that the emotional working memory training may be effective in reducing mild to moderate levels of trait anxiety.

It must be noted that potential placebo effects must be considered in the interpretation of these findings. The control group was largely inactive, and were merely told to practice healthy habits during the 20 day period. This may not have been sufficient to account for placebo effects. However, it must also be considered that these potential placebo effects were attenuated by several measures taken, such as ensuring that the experimental group were aware of the presence of a control group, but were not made aware of which condition they were assigned to. Moreover, it was ensured in the recruitment process that participants would not communicate with one another, identify one another, or disclose any details of their condition. This may have mitigated some treatment expectations. Additionally, in the mediation analysis, working memory capacity gains was used as a predictor as opposed to a categorical factor such as having undergone training or not, in order to reduce the impact of the aforementioned placebo effects. As the measure for working memory capacity was an objective and standardized measure of ability with sound test-retest reliability and utilized alternate forms (Unsworth et al., 2005), it is less susceptible to placebo and practice or training effects. Because these gains were used as a predictor for anxiety reduction in the mediation analysis, placebo effects were attenuated. Nevertheless, the risk of placebo effects cannot be entirety mitigated.

With regard to emotion regulation, while scores significantly increased from pre-training to post-training for the experimental group, significant increases were found for the control group as well. This indicates that increases of scores on the emotional regulation scale on both groups may possibly be influenced by test-retest bias and/or other external factors. Moreover, the interaction effect and main effect of the working memory training were not significant. That said, there is a lack of evidence to support that the working memory training was effective in improving emotion regulation as measured by the ERQ.

In the mediation analysis, working memory gains did not have a significant relationship with emotion regulation. Emotion regulation gains were found to be related to trait anxiety reduction, which reinforced that emotion regulation plays a vital role in diminishing trait anxiety (Cisler et al., 2010), however, these gains are not associated to the working memory training and again, have likely been brought about by expectations (Miller et al., 2009; Darragh et al., 2016). That said, emotion regulation, as measured by the ERQ, did not prove to mediate the relationship between working memory capacity gains and anxiety reduction.

However, it is possible that decreases in anxiety may in fact be related to specific emotion regulation mechanisms being trained. Among the emotion regulation mechanisms updating, distraction, and concentration, only updating was directly measured by the ERQ. Emotion suppression, which is another domain measured by the ERQ, is theoretically believed to be dependent on distraction and concentration mechanisms (Gross and John, 2003; Enebrink et al., 2013). However, emotion suppression does not directly represent these two mechanisms. Moreover, the working memory training targeted mainly attention control mechanisms subsumed by working memory (Miyake et al., 2000; Sari et al., 2015; Beloe and Derakshan, 2019). This was done by training participants to shift their attention away from irrelevant emotionally-charged stimuli, and to focus their attention on task-relevant information. These attention control mechanisms, which support the emotion regulation strategies of distraction and concertation (Schmeichel et al., 2008; Gyurak et al., 2012; Opitz et al., 2012; Berti, 2016), were centrally targeted by the working memory training, but were not directly measured by the ERQ. Their impact on anxiety reduction may therefore have been significant, which is supported by working memory capacity gains being associated with anxiety reduction. However, this may not have been captured by the ERQ as a mediator. These findings also suggest that while the emotion regulation mechanism of updating might be useful in decreasing levels of trait anxiety, this process may not necessarily be targeted by working memory training, reinforcing the conclusions made by Lee and Xue (2018) in their study.

### Implications

Firstly, the study used a measure of working memory capacity that is not training-specific, to emphasize the role of attentional control in working memory more broadly. It therefore suggests that some forms of working memory training may be effective in improving working memory capacity. These findings may be useful to consider in neurorehabilitation and cognitive remediation programs.

The study also suggests that working memory training could be helpful in reducing mild to moderate levels of trait anxiety. The possibility of utilizing the training program as a supplement to clinical interventions can be explored in these populations. The training might also be considered for individuals with mild to moderate levels of anxiety who are looking for an inexpensive and readily-available training program that can be easily accessed from their own homes when face-to-face therapy is not convenient or possible.

The study also contributes to cognitive theories of anxiety that suggest that attention bias and control issues are a cause for anxious experiences (Aikins and Craske, 2001). The study’s results support findings of other researchers regarding the role of attentional control in reducing anxiety (Sari et al., 2015; Beloe and Derakshan, 2019), thereby reinforcing this theory. Moreover, the study builds on this framework by examining how specific attentional control mechanisms (i.e., shifting, sustained attention) are translated into emotion regulation strategies (i.e., distraction and concentration), which may be dependent on working memory capacity.

### Limitations and Recommendations

As discussed, the study is limited by a lack of a placebo condition. Future research may make use of a placebo group in order to ascertain that changes in scores were not due to any sort of expectations about the program (Miller et al., 2009; Darragh et al., 2016). Meta-analytic research can also combine studies that have used a placebo condition with the information derived from this research.

Another limitation of the study is a that the ERQ did not take the emotion regulation mechanisms of distraction and concentration into account. Future studies may strive to utilize an emotion regulation measure that includes these mechanisms. The emotion regulation tool utilized by Xiu et al. (2018), for example, would be a viable option.

Another limitation is the baseline cut-off score for trait anxiety reflecting only normal levels of trait anxiety. While the baseline mean of the experimental group did reflect clinical levels of trait anxiety, because some participants within this sample had sub-clinical levels of anxiety at baseline, caution must be exercised when determining how effective the training would be in individuals with more severe levels of trait anxiety. It is recommended that future research examine the efficacy of the working memory training in purely clinical populations, or with populations that have more severe levels of trait anxiety, while controlling for confounders such as the use of other psychological and pharmaceutical interventions.

It would also be interesting for future studies to explore somatic manifestations of trait anxiety by utilizing measures of anxiety such as the Beck Anxiety Scale-Trait, which focuses more on physiological experiences of trait anxiety. Lastly, long term impacts of the emotional working memory training on trait anxiety can be explored.

## Data Availability Statement

The raw data supporting the conclusions of this article will be made available by the authors, without undue reservation.

## Ethics Statement

The studies involving human participants were reviewed and approved by the University Research Ethics Office, Ateneo de Manila University. The patients/participants provided their written informed consent to participate in this study.

## Author Contributions

GV and WT conceptualized the research together. GV was responsible for writing the manuscript, collecting the data, running statistical analysis, and interpreting the data in the conclusion and discussions section. WT was responsible for determining which articles to select for the literature review, determining the methods and materials, as well as which statistical analysis to run and also assisted in writing sections of the manuscript and editing the final output. Both authors contributed to the article and approved the submitted version.

## Conflict of Interest

The authors declare that the research was conducted in the absence of any commercial or financial relationships that could be construed as a potential conflict of interest.

## References

[B1] Aikins D. E. Craske M. G. (2001). Cognitive theories of generalized anxiety disorder. *Psychiatr. Clin. North Am.* 24 57–74. 11225509 10.1016/s0193-953x(05)70206-9

[B2] Beloe P. Derakshan N. (2019). Adaptive working memory training can reduce anxiety and depression vulnerability in adolescents. *Dev. Sci.* 23:e12831. 10.1111/desc.12831 30927316

[B3] Berti S. (2016). Switching attention within working memory is reflected in the P3a component of the human event-related brain potential. *Front. Hum. Neurosci.* 9:701. 10.3389/fnhum.2015.00701 26779009 PMC4701918

[B4] Cisler J. M. Olatunji B. O. Feldner M. T. Forsyth J. P. (2010). Emotion regulation and the anxiety disorders: an integrative review. *J. Psychopathol. Behav. Assess.* 32 68–82. 20622981 10.1007/s10862-009-9161-1 PMC2901125

[B5] Culpepper L. Conner K. (2004). Effective recognition and treatment of generalized anxiety disorder in primary care. *Prim. Care Companion J. Clin. Psychiatry* 6 35–41. 10.4088/pcc.v06n0107 15486599 PMC427612

[B6] Darragh M. Yow B. Kieser A. Booth R. J. Kydd R. R. Consedine N. S. (2016). A take-home placebo treatment can reduce stress, anxiety and symptoms of depression in a non-patient population. *Aust. N. Z. J. Psychiatry* 50 858–865. 10.1177/0004867415621390 26681262

[B7] Dennis T. A. Hajcak G. (2009). The late positive potential: a neurophysiological marker for emotion regulation in children. *J. Child Psychol. Psychiatry* 50 1373–1383. 10.1111/j.1469-7610.2009.02168.x 19754501 PMC3019134

[B8] Ecker U. K. Lewandowsky S. Oberauer K. Chee A. E. (2010). The components of working memory updating: an experimental decomposition and individual differences. *J. Exp. Psychol. Learn. Mem. Cogn.* 36 170–189. 10.1037/a0017891 20053053

[B9] Enebrink P. Björnsdotter A. Ghaderi A. (2013). The emotion regulation questionnaire: psychometric properties and norms for Swedish parents of children aged 10-13 years. *Eur. J. Psychol.* 9 289–303. 10.5964/ejop.v9i2.535

[B10] Engle R. W. (2002). Working memory capacity as executive attention. *Curr. Dir. Psychol. Sci.* 11 19–23. 10.1111/1467-8721.00160

[B11] Eysenck M. W. Derakshan N. Santos R. Calvo M. G. (2007). Anxiety and cognitive performance: attentional control theory. *Emotion* 7 336–353. 10.1037/1528-3542.7.2.336 17516812

[B12] Foti D. Hajcak G. (2008). Deconstructing reappraisal: descriptions preceding arousing pictures modulate the subsequent neural response. *J. Cogn. Neurosci.* 20 977–988. 10.1162/jocn.2008.20066 18211235

[B13] Franken I. (2014). *Tackling Depression and Anxiety: A Working Memory Intervention ClinicalTrials.gov.* Available online at: https://clinicaltrials.gov/ct2/show/NCT02119923 (accessed April 22, 2014).

[B14] Gellman M. D. (2012). *Encyclopedia of Behavioral Medicine.* New York, NY: Springer.

[B15] Gross J. J. John O. P. (2003). Individual differences in two emotion regulation processes: implications for affect, relationships, and well-being. *J. Pers. Soc. Psychol.* 85 348–362. 10.1037/0022-3514.85.2.348 12916575

[B16] Gyurak A. Goodkind M. S. Kramer J. H. Miller B. L. Levenson R. (2012). Executive functions and the down-regulation and up-regulation of emotion. *Cogn. Emot.* 26 103–118. 10.1080/02699931.2011.557291 21432634 PMC3155745

[B17] Hadwin J. A. Richards H. J. (2016). Working memory training and CBT reduces anxiety symptoms and attentional biases to threat: a preliminary study. *Front. Psychol.* 7:47. 10.3389/fpsyg.2016.00047 26869956 PMC4735443

[B18] Kashdan T. B. Zvolensky M. J. McLeish A. C. (2008). Anxiety sensitivity and affect regulatory strategies: individual and interactive risk factors for anxiety-related symptoms. *J. Anxiety Disord.* 22 429–440. 10.1016/j.janxdis.2007.03.011 17449221 PMC2673808

[B19] Kensinger E. A. Corkin S. (2003). Effect of negative emotional content on working memory and long-term memory. *Emotion* 3 378–393. 10.1037/1528-3542.3.4.378 14674830

[B20] Lee T. W. Xue S. W. (2018). Does emotion regulation engage the same neural circuit as working memory? A meta-analytical comparison between cognitive reappraisal of negative emotion and 2-back working memory task. *PLoS One* 13:e0203753. 10.1371/journal.pone.0203753 30212509 PMC6136767

[B21] Leone de Voogd E. Wiers R. W. Zwitser R. J. Salemink E. (2016). Emotional working memory training as an online intervention for adolescent anxiety and depression: a randomised controlled trial. *Aust. J. Psychol.* 68 228–238. 10.1111/ajpy.12134 27917000 PMC5129510

[B22] Mammarella N. (2014). Is emotional working memory training a new avenue of AD treatment? A review. *Aging Dis.* 5 35–40. 10.14336/ad.2014.050035 24490115 PMC3901612

[B23] Miller F. G. Colloca L. Kaptchuk T. J. (2009). The placebo effect: illness and interpersonal healing. *Perspect. Biol. Med.* 52 518–539. 10.1353/pbm.0.0115 19855122 PMC2814126

[B24] Miyake A. Friedman N. P. Emerson M. J. Witzki A. H. Howerter A. Wager T. D. (2000). The unity and diversity of executive functions and their contributions to complex “frontal lobe” tasks: a latent variable analyses. *Cogn. Psychol.* 41 49–100. 10.1006/cogp.1999.0734 10945922

[B25] Onraedt T. Koster E. H. (2014). Training working memory to reduce rumination. *PLoS One* 9:e90632. 10.1371/journal.pone.0090632 24595102 PMC3940909

[B26] Opitz C. Gross J. Urry L. (2012). Selection, optimization, and compensation in the domain of emotion regulation: applications to adolescence, older age, and major depressive disorder. *Soc. Pers. Psychol. Compass* 6 142–155. 10.1111/j.1751-9004.2011.00413.x

[B27] Pe M. L. Raes F. Kuppens P. (2013). The cognitive building blocks of emotion regulation: ability to update working memory moderates the efficacy of rumination and reappraisal on emotion. *PLoS One* 8:e69071. 10.1371/journal.pone.0069071 23874872 PMC3715480

[B28] Redick T. S. Broadway J. M. Meier M. E. Kuriakose P. S. Unsworth N. Kane M. J. (2012). Measuring working memory capacity with automated complex span tasks. *Eur. J. Psychol. Assess.* 28 164–171. 10.1027/1015-5759/a000123

[B29] Roughan L. Hadwin J. A. (2011). The impact of working memory training in young people with social, emotional and behavioural difficulties. *Learn. Individ. Differ.* 21 759–764. 10.1016/j.lindif.2011.07.011

[B30] Sari B. A. Koster E. H. Pourtois G. Derakshan N. (2015). Training working memory to improve attentional control in anxiety: a proof-of-principle study using behavioral and electrophysiological measures. *Biol. Psychol.* 121 203–212. 10.1016/j.biopsycho.2015.09.008 26407521

[B31] Schmeichel B. J. Volokhov R. N. Demaree H. A. (2008). Working memory capacity and the self-regulation of emotional expression and experience. *J. Pers. Soc. Psychol.* 95 1526–1540. 10.1037/a0013345 19025300

[B32] Schweizer S. Grahn J. Hampshire A. Mobbs D. Dalgleish T. (2013). Training the emotional brain: improving affective control through emotional working memory training. *J. Neurosci.* 33 5301–5311. 10.1523/jneurosci.2593-12.2013 23516294 PMC6704999

[B33] Schweizer S. Hampshire A. Dalgleish T. (2011). Extending brain-training to the affective domain: increasing cognitive and affective executive control through emotional working memory training. *PLoS One* 6:e24372. 10.1371/journal.pone.0024372 21949712 PMC3176229

[B34] Smith M. A. (2016). *Dual N-Back Tutorial: Classic and Emotional Dual N-Back with Dual N-Back Pro*. Available online at: https://dual-n-back-pro.com/

[B35] Spielberger C. D. (1989). *State-Trait Anxiety Inventory: Bibliography*, 2nd Edn. Palo Alto, CA: Consulting Psychologists Press.

[B36] Spielberger C. D. Gorsuch R. L. Lushene R. Vagg P. R. Jacobs G. A. (1983). *Manual for the State-Trait Anxiety Inventory.* Palo Alto, CA: Consulting Psychologists Press.

[B37] Takeuchi H. Taki Y. Nouchi R. Hashizume H. Sekiguchi A. Kotozaki Y. (2014). Working memory training improves emotional states of healthy individuals. *Front. Syst. Neurosci.* 8:200. 10.3389/fnsys.2014.00200 25360090 PMC4199268

[B38] Turner M. L. Engle R. W. (1989). Is working memory capacity task dependent? *J. Mem. Lang.* 28 127–154. 10.1016/0749-596x(89)90040-5

[B39] Unsworth N. Heitz R. P. Schrock J. C. Engle R. W. (2005). An automated version of the operation span task. *Behav. Res. Methods* 37 498–505. 10.3758/bf03192720 16405146

[B40] Veerapa E. Grandgenevre P. El Fayoumi M. (2020). Attentional bias towards negative stimuli in healthy individuals and the effects of trait anxiety. *Sci. Rep.* 10:11826. 10.1038/s41598-020-68490-5 PMC7367300 32678129

[B41] Vitasari P. Wahab M. N. Herawan T. Othman A. Sinnadurai S. K. (2011). Re-test of state trait anxiety inventory (STAI) among engineering students in Malaysia: reliability and validity tests. *Procedia Soc. Behav. Sci.* 15 3843–3848. 10.1016/j.sbspro.2011.04.383

[B42] Wanmaker S. Geraerts E. Franken I. H. (2015). A working memory training to decrease rumination in depressed and anxious individuals: a double-blind randomized controlled trial. *J. Affect. Disord.* 175 310–319. 10.1016/j.jad.2014.12.027 25661397

[B43] Wanmaker S. Hopstaken J. F. Asselbergs J. Geraerts E. Franken I. H. (2014). Decreasing dysphoric thoughts by a working memory training: a randomized double-blind placebo-controlled trial. *J. Depress. Anxiety* 3:165.

[B44] Wilhelm O. Hildebrandt A. Oberauer K. (2013). What is working memory capacity, and how can we measure it? *Front. Psychol.* 4:433. 10.3389/fpsyg.2013.00433 23898309 PMC3721021

[B45] Williams J. M. G. Watts F. N. MacLeod C. Mathews A. (1988). *Cognitive Psychology and Emotional Disorders.* Chichester: Wiley.

[B46] Xiu L. Wu J. Chang L. Zhou R. (2018). Working memory training improves emotion regulation ability. *Sci. Rep.* 8:15012. 10.1038/s41598-018-31495-2 PMC6177433 30301906

